# A new species of *Rhododendron* (Ericaceae) from Guizhou, China

**DOI:** 10.3897/phytokeys.146.51342

**Published:** 2020-05-08

**Authors:** Xiao-Yong Dai, Cheng-Hua Yang, Bing Yang, Pu Chen, Yong-Peng Ma

**Affiliations:** 1 Guizhou Academy of Forestry, Guiyang 550009, Guizhou, China Guizhou Academy of Forestry Guiyang China; 2 Puding Forestry and Grassland Administration, Puding 562100, Guizhou, China Puding Forestry and Grassland Administration Puding China; 3 Yunnan Key Laboratory for Integrative Conservation of Plant Species with Extremely Small Populations, Kunming Institute of Botany, Chinese Academy of Sciences, Kunming 650201, Yunnan, China Kunming Institute of Botany, Chinese Academy of Sciences Kunming China

**Keywords:** China, Ericaceae, Guizhou, *Rhododendron*, *R.pudingense*, *Tsutsusi*

## Abstract

A new species of the *Rhododendron* (Ericaceae) in subgen. Tsutsusisect.Tsutsusi from Puding county of Guizhou, China, is described and illustrated. The new species, *R.pudingense* X.Y. Dai, C.H. Yang & Y.P. Ma, is similar to *R.myrsinifolium* Ching ex Fang et M. Y. He and *R.minutiflorum* Hu, but it can be easily distinguished by its length and being pubescent on inner surface of corolla tube, sparse hairs below the middle of filament and the glabrous style.

## Introduction

*Rhododendron* Linn., the largest genus of woody plants in the Ericaceae (Chamberlain et al. 1996, Ma et al. 2010), consists of more than 1000 species (Wu et al. 2005). China is recognized as one of the main distribution and diversity centers for the genus *Rhododendron* (Geng 2014). There have been a number of new species reported from China in recent years (Liao et al. 2015, Ma et al. 2015, Cai et al. 2016), and there are now more than 600 *Rhododendron* species known from the country (Tian et al. 2019).

Sleumer (1949, 1980) classified RhododendronsubgenusTsutsusi Pojarkova into three sections. Based on molecular evidence from the analysis of ITS, combined with features of leaf anatomic structure and vessels, Liu (2007) divided RhododendronsubgenusTsutsusi into 74 species and two subspecies as well as 13 varieties and one form. More recently, Geng (2014) revised RhododendronsubgenusTsutsusi into two sections (*Tsutsusi* and *Brachycalyx*) recognizing in the process 58 species (figures include both variety and form), which accounts for 72.5% all over the world.

The Yunnan-Kweichow Plateau is famous for its plant diversity in China (Huang et al. 2018). However, unlike Yunnan where there have been many explorations for plants, large areas of Guizhou remain to be investigated (Huang et al. 2018). During May 2018 fieldwork in the Puding County of the Guizhou Province discovered an unknown *Rhododendron* species. The following year, during April 2019 the same *Rhododendron* was also discovered again in the Wangmo County of the same province. Examination of *Rhododendron* specimens and relevant literature for the genus from all over the world revealed that the morphological characters of the potentially new species do not fit any known species of *Rhododendron* (Liu 2007, Geng 2014, Liao et al. 2015, Ma et al. 2015, Cai et al. 2016, Tian et al. 2019). Hence, we concluded that the Puding and Wangmo County, Guizhou Province *Rhododendron* specimens represent a species new to science, which we formally described here.

## Materials and methods

Information on living plants and habitats was obtained from field investigations in 2018 and in 2019. Species descriptions and measurements were obtained from field notes and dried herbarium specimens. Then, the specimens were identified by a thorough literature examination (Wu et al. 2005, Geng 2014) and compared with type specimen images available online (JSTOR Global Plants, http://plants.jstor.org/). We also examined herbarium specimens from KUN, GF and from the online tools CVH (http://www.cvh.ac.cn/).

## Results

### Taxonomic treatment

#### Rhododendron pudingense

Taxon classificationPlantaeEricalesEricaceae

X.Y. Dai, C.H. Yang & Y.P. Ma. sp. nov.

0F5D571A-9B65-5D26-A2E1-0F7A016C3D48

urn:lsid:ipni.org:names:77209564-1

Figs 1 , 2

##### Type.

China. Guizhou: Puding County, Machang town, Longjinshan, 26°17'34.08"N, 105°35'20.04"E, altitude 1400 m alt., 7 May 2018, *XiaoyongDai 180507112* (fl., Holotype GF!, isotypes KUN!, PE!).

##### Description.

*Shrubs* evergreen. *Branches* subverticillate, young shoots with coarsely strigose, hairs flat. *Leaves* hard leathery, crowded at branch top, obovate to obovate-elliptic, 8–20 × 5–10 mm; apex obtuse, obtusely pointed or mucronate, base cuneate, margin slightly revolute; abaxial surface sparsely with coarsely brown strigose except for midvein; adaxial surface green, margin sparsely with coarsely strigose at first, and then fall off gradually except for midvein; adaxial abaxial surface greyish white, while yellow-white when dry; midrib and lateral veins concaved on abaxial surface and projecting on abaxial surface, lateral veins inconspicuous visible on both sides, anastomosing margin. *Petioles* 2–4 mm at length, densely with coarsely dark brown strigose. *Floral bud* ovoid, paleta ovoid, 7–10 × 4–6 mm, outer surface ridge coarsely strigose, margin villous. *Inflorescence* terminal, 4–6-flowered. *Pedicel* 4–10 mm at length, densely coarsely strigose. *Calyx* slightly 5-lobed, delta, sinuate, 1 × 1.5 mm, outer side coarsely strigose, margin densely. *Corolla* funnel, bilaterally symmetric, 15–20 mm at length; tube cylindric, inner surface slightly puberulent, 8–11 × 2–3 mm; lobes 5, apex obtusely pointed or mucronate, equal, 6–9 × 4 mm. *Stamens* 5, subequal, exerted, 15–28 mm at length; filaments pink, slightly puberulent; anthers purple; ovary ovoid, 3.5 mm at length, densely coarsely strigose. *Style* ca. 20–28 mm at length, longer than parts of stamens, purplish red at middle and lower part, glabrous; stigmas capitate, pink. *Capsule* oblong, 5 mm at length, coarsely strigose.

**Figure 1.**
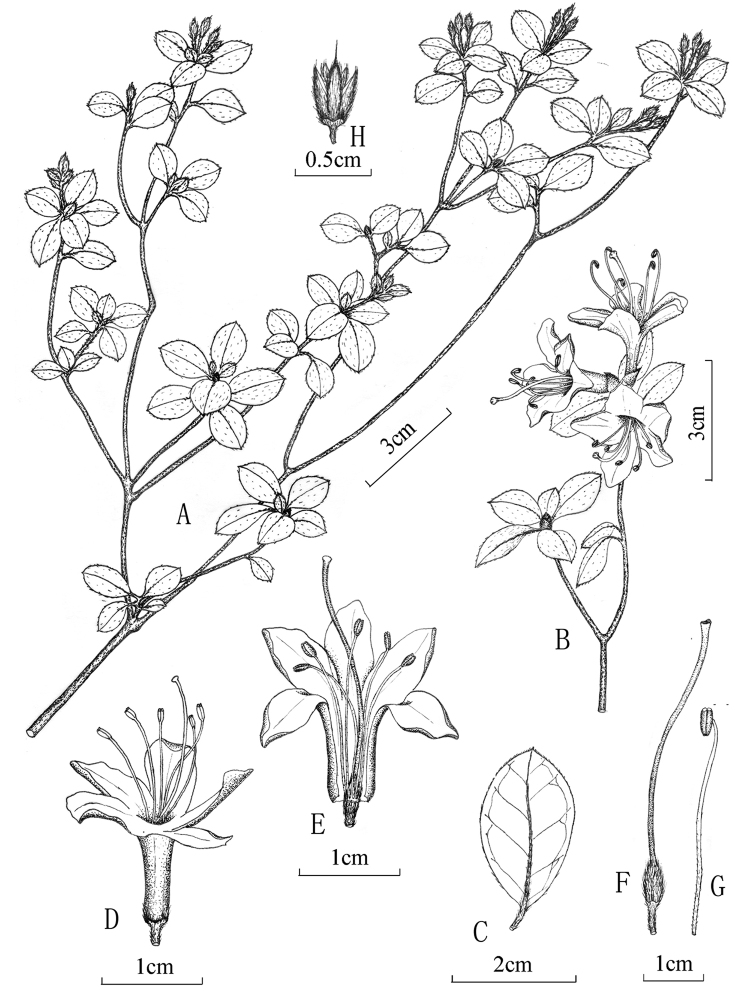
*Rhododendronpudingense* X.Y. Dai & C.H. Yang, sp. nov. **A** fruiting branch **B** flowering branch **C** leaf abaxial **D** flowers **E** side view of flower **F** stigma and ovary **G** stamen **H** capsule.

##### Etymology.

The specific epithet is named after the type locality Puding County, in which this plant was first discovered.

##### Vernacular name.

Chinese mandarin: pǔ dìng dù juān (普定杜鹃)

**Phenology.** This new species has been observed in flowering from mid-April to early May and fruiting from early May to October.

##### Distribution and habitat.

So far, this species is only known from the type locality (Pudding County) and from the Wangmo County, Guizhou Province, southwest China. At these locations *Rhododendronpudingense* grows in evergreen and deciduous broad leaved mixed open forests within the rock cracks of limestone hills, at an elevation of 1300 m to 1400 m.

##### Conservation status.

*Rhododendronpudingense* is currently known only from two locations, Puding County and Wangmo County (Guizhou Province, China) with a combined population estimated at 100 individuals, and an area of occupancy (AOO) of <500 kilometers. Within this species known range the populations are severely fragmented and occupy a vegetation association in decline; we therefore propose to treat it as [EN B2ab(iii); D] in accordance to the IUCN Red List Categories and Criteria version 13 (IUCN Standards and Petitions Subcommittee 2017).

##### Additional specimens examined.

China. **Guizhou**: Puding County, Machang Town, Longjinshan, altitude Longjinshan, 26°17'34.08"N, 105°35'20.04"E, 1400 m alt., 20 August 2018, *Xiaoyong Dai 18082201* (fr., paratype GF!); China. **Guizhou**: Wangmo County, Mashan Town, Heidong, 25°14'21.05"N, 106°22'13.73"E, 1300 m alt., 10 April 2019, *Xiaoyong Dai* &*Jianghua HUANG 19041002* (fl., paratype GF!).

**Figure 2.**
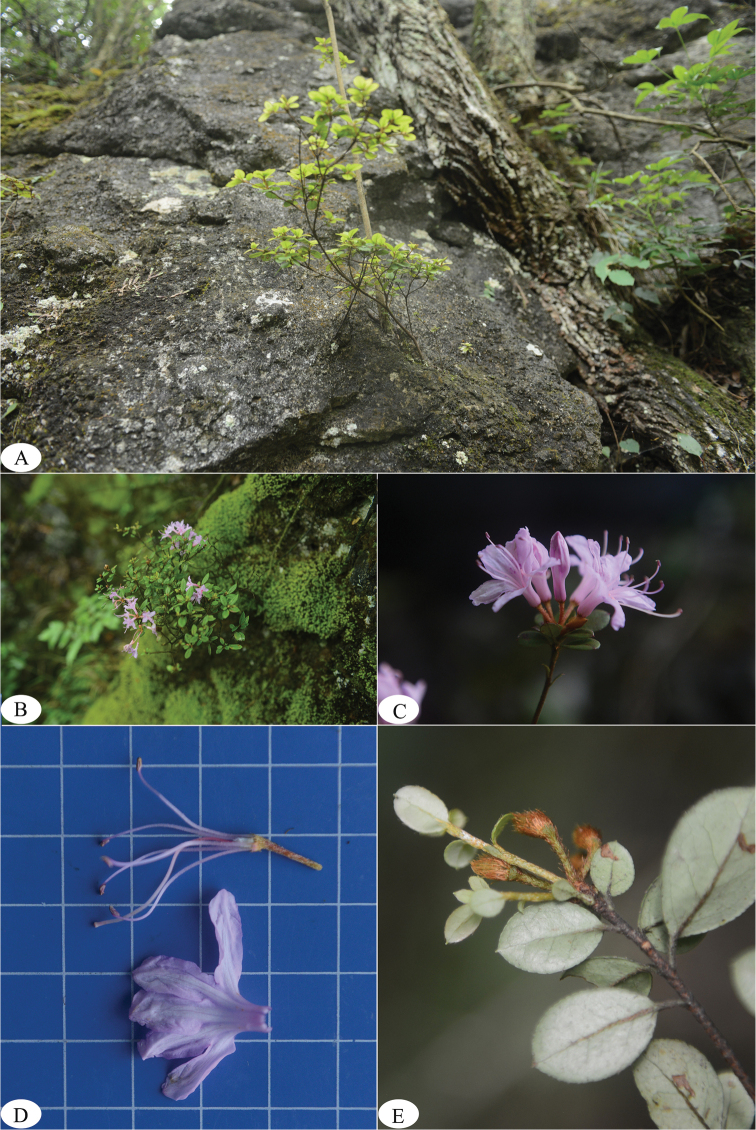
*Rhododendronpudingense* X.Y. Dai & C.H. Yang, sp. nov. **A** habitat **B** one flowering plant **C** inflorescence **D** flower viewed from different angles as well as stigma and filaments **E** fruit branch.

##### Relationships.

The new species is morphologically similar to *R.myrsinifolium* and *R.minutiflorum* (Table 1), however, *R.pudingense* can be easily distinguished from these species by examination of the corolla tube, stamens and styles. Notably, *R.pudingense* has a longer corolla tube (8–11 mm long) than *R.myrsinifolium* and *R.minutiflorum*, whose corolla tube lengths are both < 4 mm (Table 1). In addition, *R.pudingense* has glabrous styles whereas the lower part of style in *R.myrsinifolium* and *R.minutiflorum*, is furnished with coarsely appressed setose hairs or sparsely shortly glandular-hairy (Table 1). Furthermore, the stamens of *R.pudingense* are 15–28 mm long and slightly puberulent below the middle of filament, where those of *R.myrsinifolium*, are 12–14 mm long and glabrous, and the stamens of *R.minutiflorum* are 7 mm long and puberulent in their lower portion (Table 1).

**Table 1.** Diagnostic characters for *Rhododendronpudingense* and closely related species.

**Characters**	***R.myrsinifolium***	***R.pudingense***	***R.minutiflorum***
Leaf shape	elliptic, rarely obovate	obovate to obovate-elliptic	oblong to obovate or broadly ovate
Leaf length	6–8×3–5 mm	8–20 × 5–10 mm	8–15 × 3–5 mm
Leaf margin	red-brown punctate glands	no glands	no glands
Corolla tube length	4 mm long	8–11 mm long	3 mm long
Corolla tube surface	glabrous in both sides	outer surface glabrous whereas inner surface puberulent	outer surface reddish glandular-hairy and inner surface puberulent
Stamen length	12–14 mm	15–28 mm	7 mm long
Filament	glabrous	slightly puberulent below the middle of filament	puberulent at the lower part
Style length	12–15mm	20–28 mm	8 mm long
Style covers	coarsely appressed setose at the lower part	glabrous	Sparsely shortly glandular-hairy below

## Supplementary Material

XML Treatment for Rhododendron pudingense
